# Regional growth patterns of fetal corpus callosum at 18–32 weeks' gestation assessed by three‐dimensional transvaginal neurosonography

**DOI:** 10.1002/uog.70334

**Published:** 2026-10-21

**Authors:** Y. Chen, Y. Liao, Q. Zhang, M. Machida, K. Uenishi, J. Lin, R. K. Pooh, L. C. Poon

**Affiliations:** ^1^ Department of Obstetrics and Gynaecology, Prince of Wales Hospital The Chinese University of Hong Kong Shatin Hong Kong SAR China; ^2^ Department of Ultrasound, Shenzhen Maternity and Child Healthcare Hospital, Women and Children's Medical Center Southern Medical University Shenzhen China; ^3^ Department of Obstetrics and Gynecology, Fujian Maternity and Child Health Hospital Fujian Medical University Teaching Hospital Fujian China; ^4^ Fetal Brain Center, CRIFM Prenatal Medical Clinic Osaka Japan

**Keywords:** corpus callosum, developmental biometry, fetus, gestational age, neurosonography

## Abstract

**Objectives:**

To evaluate the normal development of the fetal corpus callosum (CC) between 18 and 32 weeks' gestation using three‐dimensional (3D) transvaginal neurosonography and to assess the effect of volume contrast imaging (VCI) on the measurement of fetal CC biometry.

**Methods:**

This study included two cohorts of women with a singleton pregnancy: a prospective cohort attending the research clinic at Prince of Wales Hospital in Hong Kong SAR, China, between April 2019 and February 2025, at which 3D fetal brain volumes were acquired prospectively, and a retrospective cohort attending CRIFM Prenatal Medical Clinic in Osaka, Japan, between December 2018 and March 2025, from which archived 3D fetal brain volume datasets were retrieved. In both cohorts, 3D fetal brain volume data of the CC were acquired using transvaginal neurosonography between 18 and 32 weeks' gestation using the same acquisition protocol. Inclusion criteria were appropriately grown fetuses at 18 + 0 to 32 + 6 weeks' gestation, without suspected structural or chromosomal defects. In the midsagittal plane, using 3D volume multiplanar imaging, the CC length and total area were obtained and regional thickness at the genu, body and splenium were measured following a modified Garel's method. Additionally, the areas of five vertical CC segments defined by arithmetic fractions of the CC length following Witelson's classification (anterior one‐third, anterior midbody, posterior midbody, isthmus and splenium) were obtained. All fetal biometric measurements were performed using non‐VCI and VCI modes. Measurements obtained using non‐VCI and VCI modes were compared using the paired *t*‐test. Generalized Additive Models for Location, Scale and Shape (GAMLSS) were used to explore the relationship between all biometric measurements of the fetal CC and gestational age. The final models were used to derive percentiles and construct reference curves. Intra‐ and interobserver variability were assessed using 30 randomly selected cases.

**Results:**

A total of 956 fetuses at 18–32 weeks' gestation were included. Gestational age‐specific trajectories of fetal CC biometry were established, demonstrating distinct, non‐linear growth patterns in CC length, total area, genu, body and splenium thicknesses and the areas of five individual CC subdivisions from 18 to 32 weeks. Overall, the genu and body thicknesses increased sharply from 18 weeks to 23 weeks. Thereafter, genu thickness continued to increase gradually until 32 weeks, whereas body thickness plateaued before showing renewed growth at 31–32 weeks. The percentile curve for fetal CC total area demonstrated a steady increase from 18 to 32 weeks. Among the five subdivisions of the CC, the splenium demonstrated the most pronounced absolute area growth, reaching a median of approximately 0.40 cm^2^ by 32 weeks, with markedly greater expansion than the midbody regions throughout the second to early third trimesters. Fetal CC biometric measurements were comparable between non‐VCI and VCI measurements. Overall, the biometric measurements demonstrated high reproducibility, with most showing good‐to‐excellent intra‐ and interobserver reliability, except for moderate interobserver reliability for genu and body thicknesses.

**Conclusions:**

We have characterized region‐specific non‐linear growth patterns in fetal CC development from 18 to 32 weeks, with posterior‐predominant area expansion most pronounced in the splenium. The clinical significance of regional CC measurements remains uncertain, and the presented trajectories should be regarded as descriptions of normal developmental patterns rather than as reference charts for isolated clinical decision‐making. Longitudinal studies incorporating postnatal neurodevelopmental assessment will be required to determine their prognostic value. © 2026 The Author(s). *Ultrasound in Obstetrics & Gynecology* published by John Wiley & Sons Ltd on behalf of International Society of Ultrasound in Obstetrics and Gynecology.

## Abstract 2

Linked article: There is a comment on this article by Paladini. Click here to view the Editorial.

## INTRODUCTION

The corpus callosum (CC), the brain's largest interhemispheric commissure, contains 200–250 million contralateral axonal projections, which are essential for efficient cognitive function, bilateral cortical integration and functional coordination^1^, ^2^, ^3^. The CC is not a homogeneous structure, but rather exhibits significant regional differences in thickness. Characterizing the normal growth trajectories during pregnancy allows for a better understanding of the developmental biology of the fetal CC^4^. Paladini *et al*.^5^ presented growth charts of fetal CC length measured using transvaginal neurosonography in a large, multicenter study including 2131 individuals, addressing the critical methodological limitations of several previous studies. Consequently, their study is the currently recommended reference for measuring fetal CC length using transvaginal neurosonography.

Garel *et al*.^6^ reported reference values for the thickness of the CC in children, measuring thickness at four anatomical sites, the genu, body, isthmus and splenium, based on magnetic resonance imaging (MRI) data from children aged 1 day to 15 years. While recognizing that thickness alone is not sufficient to fully capture fetal CC developmental trajectories, we aimed to additionally characterize regional area growth patterns of fetal CC during pregnancy.

Without macroscopic anatomical landmarks that delineate distinct callosal areas in the sagittal view, a geometric scheme is needed to define the subdivisions of the CC. Researchers have previously developed geometrical schemes to subdivide the CC^7^, ^8^, ^9^, including Witelson's five‐compartment subdivision, originally derived from postnatal MRI data across pediatric populations^8^, ^9^, ^10^, ^11^, ^12^. Although alternative subdivision schemes have since been proposed, Witelson's framework was selected for use in this study owing to its operational simplicity, reproducibility across observers and prior MRI‐based evidence in pediatric populations^11^, ^13^, ^14^, ^15^. Volume contrast imaging (VCI) has been developed to enhance resolution and contrast between tissues and organs^16^, but evidence for the effect of VCI on the measurement of fetal CC biometry is sparse, with a recent study demonstrating comparable results of fetal CC length between non‐VCI and VCI modes^5^. This study aimed: (1) to evaluate normal developmental trajectories of fetal CC biometry between 18 and 32 weeks' gestation using three‐dimensional (3D) transvaginal neurosonography, integrating the modified Garel's thickness approach and Witelson's five‐subdivision area algorithm; and (2) to evaluate the effect of VCI on the measurement of fetal CC biometry.

## METHODS

This study included two cohorts of women with a singleton pregnancy: a prospective cohort attending the research clinic at Prince of Wales Hospital in Hong Kong SAR, China, at which 3D fetal brain volumes were acquired prospectively between April 2019 and February 2025; and a retrospective cohort attending a research clinic at the CRIFM Prenatal Medical Clinic in Osaka, Japan, from which archived 3D fetal brain volumes obtained between December 2018 and March 2025 using the same acquisition protocol were utilized. Inclusion criteria for the study were an appropriately grown fetus at 18 + 0 to 32 + 6 weeks' gestation, without suspected structural or chromosomal defects. All included cases were neurologically normal on hospital discharge, as confirmed by a general pediatric examination. Gestational age (GA) at the time of recruitment was determined by the first‐trimester fetal crown–rump length. Written informed consent was obtained from participants, and ethics approval for this study was provided by the respective Institutional Review Boards (CRIFM, reference No.: CRI‐IRB‐009; Joint Chinese University of Hong Kong–New Territories East Cluster Clinical Research Ethics Committee, reference No.: CRE‐2018.622).

### Three‐dimensional neurosonography

Three‐dimensional neurosonography data were obtained similarly for both the prospective and retrospective cohorts. The CC was visualized in the midsagittal plane using 3D volume multiplanar imaging, on a Voluson E10 or Expert 22 ultrasound system with a 6–12 MHz, 5–9 MHz or RIC10 (4–9 MHz) 3D transvaginal transducer (GE Healthcare, Zipf, Austria). To obtain high‐definition images of the CC, the transvaginal transducer was positioned toward the anterior fontanelle with appropriate pressure applied. A brain volume was acquired using a sweep angle of 80° to 120°, depending on the GA of the fetus. The acquisition was reviewed in real time to confirm complete visualization of all CC subdivisions. The acquired volume was displayed in three orthogonal planes, corresponding to the coronal, midsagittal and axial views. The reference view was adjusted using rotation controls until the full length of the CC was visualized in the midsagittal plane. All volume acquisitions were performed in the absence of fetal movement to minimize motion artifact. Data inclusion in the retrospective cohort was based on predefined image‐quality criteria, namely the absence of motion artifacts and adequate visualization of the midsagittal plane. Image acquisition was performed by R.K.P. (30 years of experience in transvaginal fetal neurosonography) and L.C.P. (10 years of experience in transvaginal neurosonography).

### Corpus callosum biometric measurements

Fetal CC biometric measurements of both prospective and retrospective imaging data were performed by experienced sonologists (Y.L., Q.Z.) using offline dedicated software (4DView; GE Healthcare). Y.L. and Q.Z., with 15 years and 2 years of experience, respectively, in the offline postprocessing of volume datasets, performed the reproducibility analysis. In a multiplanar display mode, we systematically aligned the orthogonal planes to a standard orientation, with the coronal plane in Plane A, the midsagittal plane in Plane B and the axial plane in Plane C. After the entire CC had been visualized in the midsagittal plane, the image was appropriately magnified to center the structure and to clearly delineate its margins. In the midsagittal plane, key anatomical landmarks, including the cavum septi pellucidi, head of the caudate nucleus, thalamus, third and fourth ventricles, parts of the midbrain, cerebellar vermis and cisterna magna, were identified.

Each volume was assessed to ensure clear visualization of the entire hypoechogenic to anechoic CC, with its borders defined by two hyperechogenic lines. The CC length, total area, regional thickness at the genu, body and splenium, and areas of each of the five CC subdivisions (Figure 1) were measured using methodology reported previously^6^, ^8^, ^17^. In the midsagittal plane, the fetal CC length was measured along a straight line from the anterior‐most inner margin of the genu to the posterior‐most inner margin of the splenium (Figure 1a).

**Figure 1:**
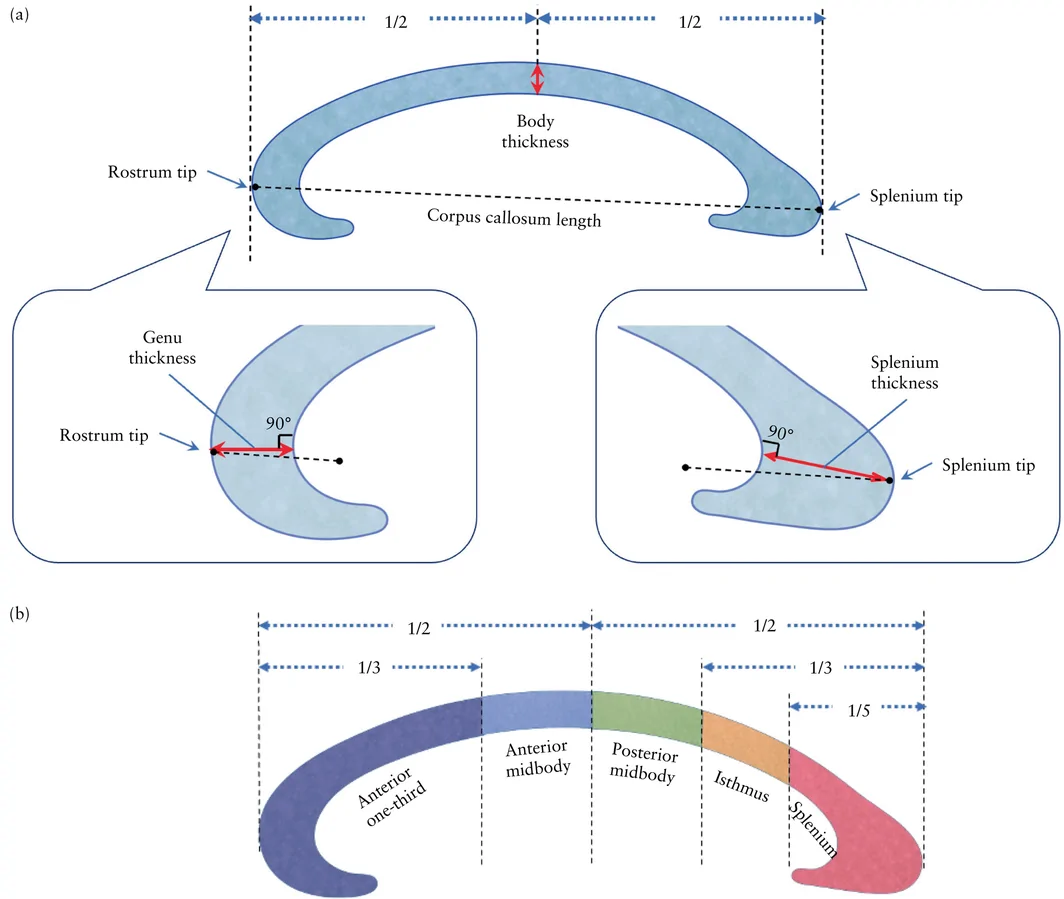
Measurements of fetal corpus callosum (CC) biometry. (a) Modified Garel's method for CC thickness measurements^6^. The CC length axis (longest chord between the rostrum tip and splenium tip on the inner (concave) border) serves as the reference. Genu thickness and splenium thickness were measured from the respective endpoint, perpendicular to the inner border tangent at that point, extending until the line intersected the outer (convex) surface (inner to inner border for measurements). Body thickness was measured at the midcallosal point (midpoint of the CC length axis), perpendicular to the local long axis of the CC. Isthmus thickness was not measured. Double‐headed arrows indicate caliper directions. (b) Five‐subdivision algorithm based on Witelson's classification^8^, in which five vertical callosal segments were defined by dividing the maximum anterior–posterior length of the CC into specific proportional fractions: the anterior one‐third, anterior midbody (anterior half minus anterior one‐third), posterior midbody (posterior half minus posterior one‐third), isthmus (posterior one‐third minus posterior one‐fifth) and splenium (posterior one‐fifth) regions.

For fetal CC thickness, direct application of Garel's method to fetal neurosonography presents some limitations owing to developmental differences in CC morphology between the prenatal and postnatal periods^6^. Specifically, isthmus thickness is defined as the narrowest point between the body and the splenium in children, with the CC–fornix junction serving as a landmark when the isthmus is not identifiable. In contrast, the fetal CC retains a tubular appearance throughout pregnancy, and the isthmic narrowing that serves as a key anatomical landmark in Garel's method is not consistently established prenatally. In light of this, the present study adopted only the thickness measurements from Garel's method that can be reliably applied to fetuses^6^. Therefore, fetal CC thickness was measured at three regions: the genu, the body and the splenium. Body thickness was measured at the midcallosal point, operationally defined as the midpoint of the CC length (half of the anterior–posterior length). The caliper was oriented perpendicular to the local long axis of the CC at this site. Although Garel *et al*.^6^ defined body thickness as being measured ‘at the level of the body’ without specifying its exact location along the body, we adopted this operational definition to ensure interobserver reproducibility across observers and institutions. This site consistently corresponds to the anatomical junction between the anterior and posterior body segments in Witelson's classification (specifically, the boundary between the ‘anterior midbody’ and the ‘posterior midbody’ (Figure 1b)), thereby providing both anatomical meaningfulness and integration with the Witelson subdivision framework^8^. With reference to the diagrammatic schema illustrated in Garel *et al*.^6^, the CC length axis was defined as the longest chord between two points on the inner (concave) border of the CC, with the anterior and posterior endpoints corresponding to the rostrum tip and the splenium tip, respectively. Genu and splenium thicknesses were then measured from the respective endpoint of the CC length axis (rostrum tip for genu thickness, splenium tip for splenium thickness), with the caliper oriented as perpendicular as possible to the inner border tangent at that endpoint, and extending until the line intersected the outer (convex) surface (inner to inner border for measurements) (Figure 1).

The total area and subdivision areas of CC were measured by manually tracing the boundary of the hypoechogenic structure. Based on Witelson's classification, five vertical CC segments following specific arithmetic fractions of the CC length were defined (Figure 1b)^8^. In particular, the CC was subdivided into five regions comprising the anterior one‐third (the combination of rostrum and genu), the anterior midbody (anterior half minus anterior one‐third), the posterior midbody (posterior half minus posterior one‐third), the isthmus (posterior one‐third minus posterior one‐fifth) and the splenium (posterior one‐fifth). To investigate the effect of VCI on fetal CC biometry, each CC was measured using VCI mode, with a slice thickness of 1 mm, and non‐VCI mode on fetal neurosonography (Figure 2). The average time required for postprocessing of the datasets by a single examiner was 8 min per case.

**Figure 2:**
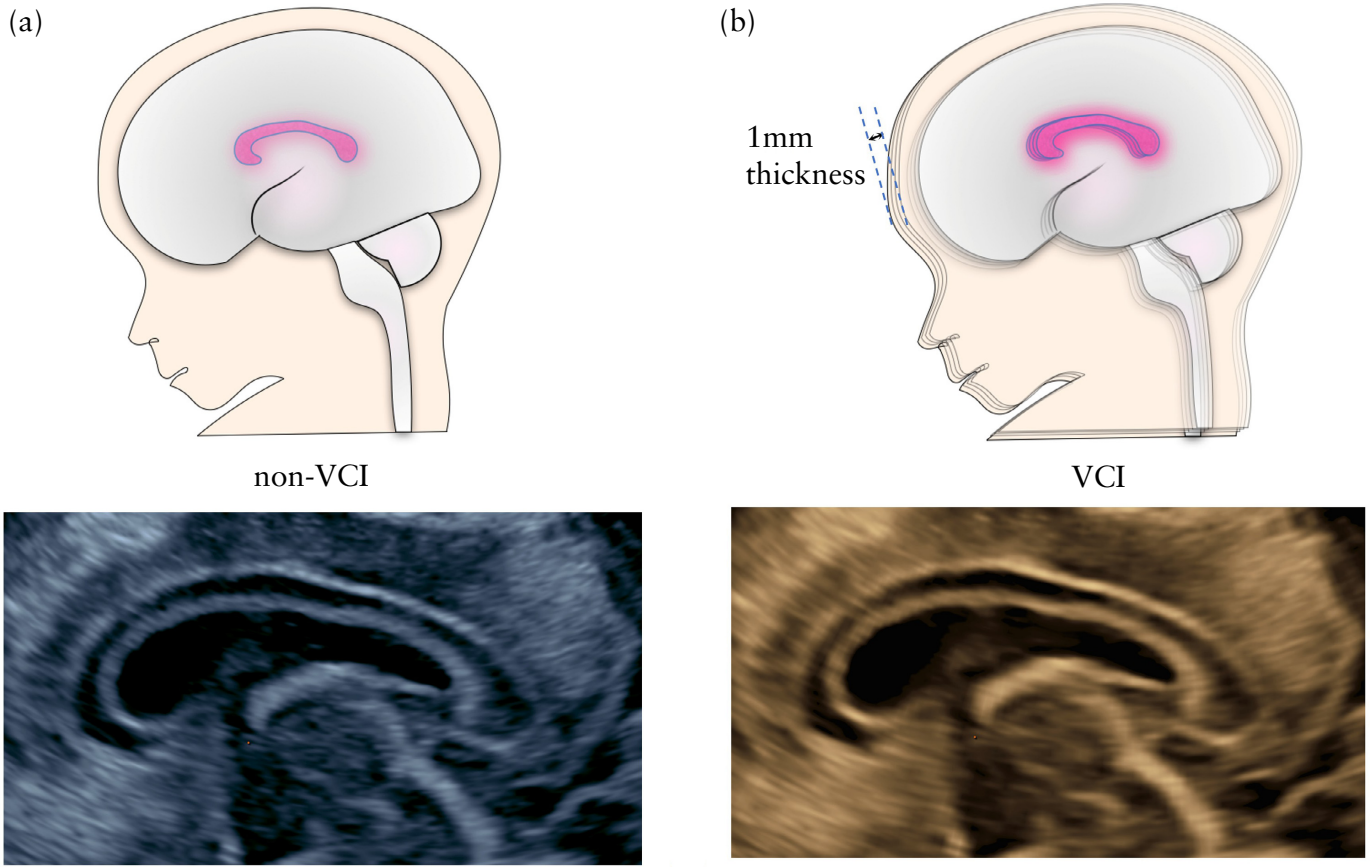
Comparison of ultrasound images of fetal corpus callosum without (a) and with (b) volume contrast imaging (VCI). Ultrasound image obtained without VCI shows standard three‐dimensional multiplanar visualization (a), while image obtained by applying VCI with a 1‐mm slice thickness projects a thin slice of tissue information, reducing speckle noise and providing clearer visualization of subtle anatomical boundaries (b).

### Statistical analysis

A paired *t*‐test was performed to compare the measurements of fetal CC biometry obtained using non‐VCI and VCI modes on neurosonography. Spearman's correlation coefficient was used to assess the association between quantitative variables. For the reproducibility analysis, intra‐ and interobserver variability were assessed using 30 randomly selected cases. The measurements of the nine variables were performed without knowledge of the GA. The two operators (Y.L., Q.Z.) were blinded to each other's measurements, and one operator (Y.L.) was blinded to their first measurements when taking the second measurements. Intraclass correlation coefficients (ICCs) were used to determine the reliability of the intra‐ and interobserver fetal CC biometric measurements. Agreement was classified as excellent (ICC ≥ 0.90), good (ICC, 0.75 to < 0.90), moderate (ICC, 0.50 to < 0.75) or poor (ICC < 0.50).

Given that a single power transformation may not adequately normalize fetal CC distributions across all GAs, a Generalized Additive Model for Location, Scale and Shape (GAMLSS)^18^ was used to explore the relationship between all biometric measurements of fetal CC and GA. The GAMLSS is highly flexible, as this approach permits the fitting of mean, coefficient of variance, skewness and kurtosis^19^. Several distributions, including the normal distribution, Box–Cox t (BCT), Box–Cox Cole and Green (BCCG) and Box–Cox power exponential (BCPE), were assessed. Best‐fit models were selected in a step‐wise manner from an initial class of models based on a normal distribution, followed by BCT, BCCG and BCPE. The goodness‐of‐fit of the models was examined using the Akaike Information Criterion. The final models were used to calculate the 2.5^th^, 5^th^, 50^th^, 95^th^ and 97.5^th^ percentiles and to construct reference curves. *P* < 0.05 was considered statistically significant. All statistical analysis was performed using R version 4.2.0 (R Foundation for Statistical Computing, Vienna, Austria).

## RESULTS

Of the 980 pregnant women initially recruited, 24 (2.45%) cases were excluded owing to poor image quality. The exclusion rates in the retrospective (*n* = 833) and prospective (*n* = 147) cohorts were 2.16% and 4.08%, respectively (*P* = 0.18). Therefore, fetal CC biometric measurements were performed and analyzed for a total of 956 cases at 18–32 weeks' gestation, comprising 815 cases from the retrospective cohort and 141 cases from the prospective cohort. The distribution of the number of cases for each GA is presented in Table S1. There were 815 (85.25%) Japanese women and 141 (14.75%) Chinese women included overall. There were no statistically significant differences in fetal CC biometry between non‐VCI and VCI measurements at any gestational week (Table S2). Fetal CC biometry is subsequently presented using non‐VCI measurements, as the non‐VCI mode is more widely available across different ultrasound platforms, thereby ensuring the practical applicability of the reference charts.

### Corpus callosum length

The relationship between the fetal CC length on non‐VCI neurosonography and advancing GA was characterized by a fourth‐degree polynomial function, with the conditional median (in cm) given by: median = 14.81412732 − (3.20422693 × GA) + (0.23400251 × GA^2^) − (0.00666237 × GA^3^ ) + (0.00006724 × GA^4^), where GA is in completed weeks (Table S3). The growth trajectory was virtually identical to that reported by Paladini *et al*.^5^ across the overlapping GA range.

### Corpus callosum thickness

Representative midsagittal views of the developing fetal CC from 18 to 32 weeks on both non‐VCI and VCI modalities are shown in Figure 3, demonstrating the progressive thickening and regional differentiation of the CC from 18 to 32 weeks. The selected distributions for fetal genu, body and splenium thicknesses were normal distribution, BCCG and BCPE distribution, respectively (Table S3). Fetal CC thickness demonstrated distinct growth in different regions from 18 to 32 weeks. The genu and body thicknesses increased sharply from 18 weeks to 23 weeks, thereafter genu thickness continued to increase gradually until 32 weeks (Table 1, Figure 4a), whereas body thickness plateaued before showing renewed growth at 31–32 weeks (Figure 4b, Table 1). Splenium thickness exhibited a sharp increase from 18 weeks to 25 weeks, followed by steady growth until 32 weeks (Figure 4c, Table 1).

**Figure 3:**
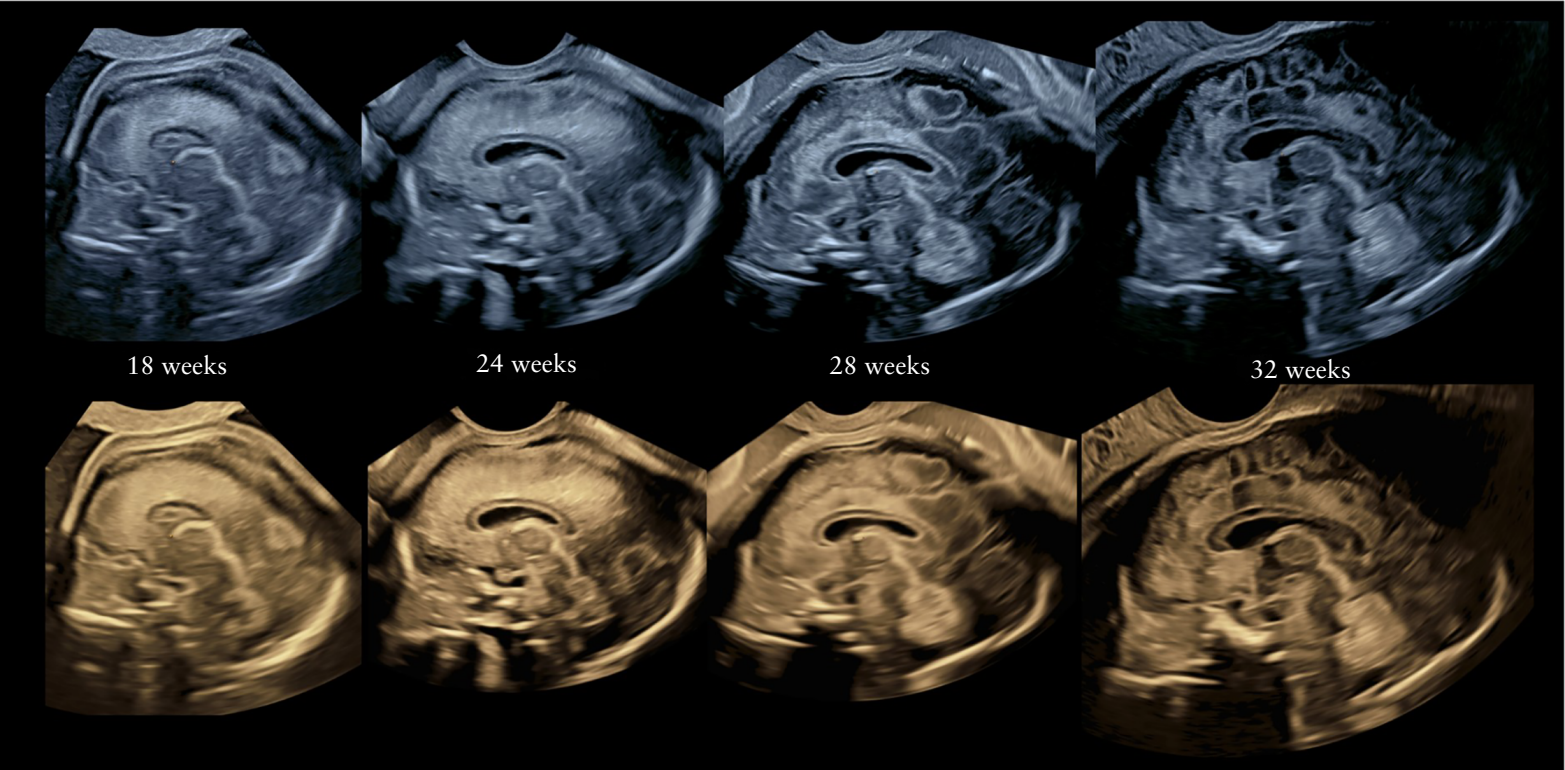
Sequential progression of fetal corpus callosum (CC) morphology from 18 to 32 weeks' gestation, showing representative midsagittal images of the fetal CC at four gestational ages (18, 24, 28 and 32 weeks) obtained by three‐dimensional transvaginal neurosonography. Upper panel demonstrates non‐volume contrast imaging (non‐VCI) and lower panel demonstrates volume contrast imaging (VCI). The CC appears thin and relatively uniform at 18 weeks, undergoes progressive thickening with emerging regional thickness variation by 24 to 28 weeks and develops distinct genu, body and splenium segments with mature morphology by 32 weeks. Both non‐VCI and VCI modalities reveal comparable morphological features, supporting the methodological equivalence of the two approaches demonstrated in the quantitative analysis.

**Figure 4:**
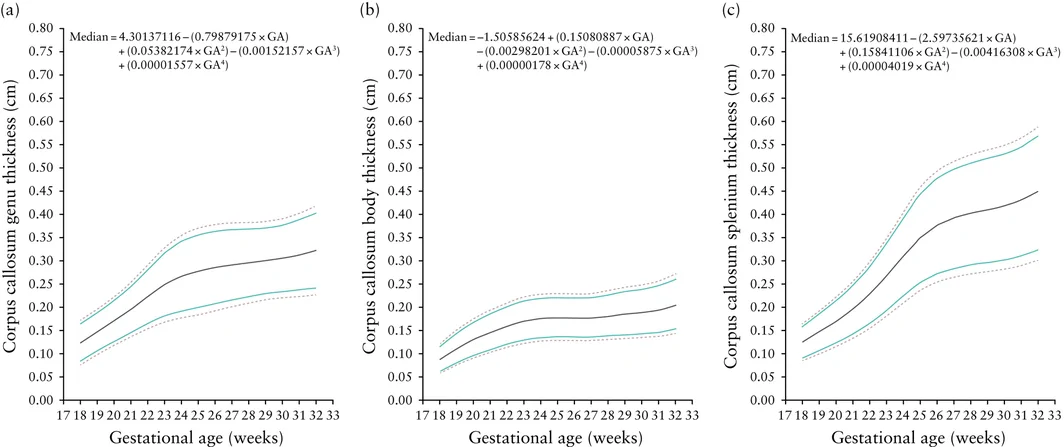
Percentile curves of fetal corpus callosum genu thickness (a), body thickness (b) and splenium thickness (c) measurements on non‐volume contrast imaging. 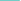, 5^th^ and 95^th^ percentiles; 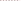, 2.5^th^ and 97.5^th^ percentiles; 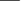, median; GA, gestational age.

**Table 1:** Estimated smoothed centiles of fetal corpus callosum (CC) biometric measurements obtained using non‐volume contrast imaging between 18 and 32 weeks' gestation

	Total area of CC (cm^2^)					CC genu thickness (cm)					CC body thickness (cm)				
GW	p2.5	p5	p50	p95	p97.5	p2.5	p5	p50	p95	p97.5	p2.5	p5	p50	p95	p97.5
18	0.071	0.078	0.122	0.170	0.178	0.075	0.083	0.123	0.164	0.172	0.058	0.062	0.087	0.115	0.121
19	0.135	0.146	0.219	0.296	0.310	0.098	0.106	0.147	0.188	0.196	0.076	0.081	0.111	0.145	0.152
20	0.205	0.221	0.317	0.419	0.437	0.118	0.126	0.170	0.214	0.222	0.091	0.097	0.131	0.169	0.176
21	0.281	0.301	0.419	0.543	0.565	0.136	0.145	0.195	0.244	0.253	0.103	0.109	0.146	0.186	0.194
22	0.361	0.384	0.525	0.673	0.699	0.154	0.165	0.223	0.280	0.291	0.114	0.121	0.159	0.202	0.211
23	0.442	0.469	0.634	0.807	0.837	0.169	0.182	0.249	0.317	0.330	0.123	0.130	0.170	0.214	0.223
24	0.518	0.549	0.739	0.938	0.973	0.178	0.192	0.267	0.343	0.357	0.128	0.135	0.176	0.220	0.229
25	0.585	0.621	0.837	1.065	1.105	0.184	0.200	0.278	0.356	0.371	0.129	0.137	0.177	0.221	0.230
26	0.642	0.683	0.928	1.186	1.231	0.193	0.208	0.286	0.364	0.379	0.129	0.136	0.177	0.220	0.229
27	0.687	0.733	1.011	1.305	1.356	0.201	0.216	0.292	0.368	0.382	0.129	0.136	0.177	0.221	0.230
28	0.723	0.775	1.091	1.425	1.484	0.209	0.223	0.296	0.369	0.383	0.131	0.139	0.181	0.227	0.237
29	0.755	0.813	1.169	1.546	1.613	0.217	0.230	0.301	0.371	0.385	0.133	0.141	0.186	0.234	0.244
30	0.784	0.849	1.245	1.667	1.741	0.221	0.234	0.306	0.377	0.391	0.135	0.143	0.189	0.239	0.249
31	0.812	0.883	1.323	1.791	1.874	0.223	0.238	0.313	0.389	0.403	0.138	0.146	0.194	0.247	0.257
32	0.836	0.915	1.401	1.922	2.014	0.227	0.242	0.323	0.403	0.418	0.144	0.154	0.205	0.261	0.273

	CC splenium thickness (cm)					CC anterior one‐third area (cm^2^)					CC anterior midbody area (cm^2^)				
GW	p2.5	p5	p50	p95	p97.5	p2.5	p5	p50	p95	p97.5	p2.5	p5	p50	p95	p97.5
18	0.085	0.091	0.125	0.158	0.164	0.023	0.026	0.042	0.059	0.062	0.003	0.005	0.015	0.025	0.026
19	0.100	0.107	0.148	0.186	0.193	0.042	0.047	0.073	0.101	0.107	0.013	0.015	0.027	0.038	0.040
20	0.115	0.123	0.169	0.213	0.221	0.066	0.072	0.109	0.148	0.156	0.022	0.025	0.038	0.052	0.055
21	0.133	0.142	0.196	0.247	0.255	0.095	0.104	0.153	0.204	0.215	0.031	0.035	0.051	0.068	0.071
22	0.154	0.165	0.228	0.287	0.297	0.127	0.138	0.200	0.265	0.277	0.040	0.044	0.064	0.084	0.088
23	0.181	0.193	0.267	0.337	0.348	0.162	0.175	0.249	0.327	0.343	0.047	0.052	0.075	0.099	0.104
24	0.210	0.225	0.310	0.392	0.405	0.193	0.208	0.293	0.383	0.401	0.052	0.057	0.084	0.110	0.115
25	0.237	0.254	0.350	0.443	0.458	0.219	0.236	0.330	0.430	0.449	0.057	0.062	0.090	0.118	0.123
26	0.255	0.273	0.377	0.477	0.494	0.241	0.260	0.362	0.469	0.490	0.061	0.067	0.095	0.124	0.129
27	0.265	0.284	0.393	0.497	0.514	0.256	0.276	0.384	0.499	0.522	0.064	0.070	0.100	0.130	0.136
28	0.272	0.292	0.403	0.510	0.528	0.267	0.288	0.403	0.525	0.549	0.068	0.074	0.106	0.137	0.143
29	0.277	0.297	0.411	0.520	0.538	0.277	0.299	0.422	0.553	0.578	0.072	0.078	0.112	0.145	0.152
30	0.282	0.302	0.418	0.529	0.548	0.286	0.310	0.442	0.581	0.608	0.075	0.082	0.118	0.154	0.161
31	0.290	0.311	0.430	0.544	0.563	0.293	0.319	0.460	0.609	0.638	0.078	0.086	0.125	0.164	0.171
32	0.302	0.324	0.449	0.568	0.588	0.299	0.327	0.477	0.636	0.668	0.083	0.091	0.133	0.175	0.183

	CC posterior midbody area (cm^2^)					CC isthmus area (cm^2^)					CC splenium area (cm^2^)				
GW	p2.5	p5	p50	p95	p97.5	p2.5	p5	p50	p95	p97.5	p2.5	p5	p50	p95	p97.5
18	0.009	0.010	0.017	0.026	0.029	0.007	0.009	0.015	0.023	0.025	0.012	0.014	0.026	0.041	0.045
19	0.017	0.019	0.029	0.043	0.047	0.014	0.016	0.026	0.039	0.041	0.025	0.028	0.049	0.075	0.080
20	0.025	0.027	0.041	0.059	0.064	0.021	0.023	0.038	0.054	0.057	0.040	0.045	0.073	0.108	0.115
21	0.033	0.036	0.053	0.074	0.079	0.028	0.031	0.049	0.068	0.073	0.057	0.063	0.098	0.142	0.151
22	0.040	0.044	0.063	0.087	0.093	0.035	0.039	0.059	0.081	0.086	0.074	0.081	0.125	0.178	0.189
23	0.047	0.052	0.073	0.099	0.105	0.041	0.045	0.067	0.092	0.097	0.092	0.101	0.154	0.218	0.231
24	0.054	0.058	0.081	0.109	0.116	0.044	0.048	0.072	0.099	0.104	0.111	0.122	0.185	0.260	0.276
25	0.059	0.064	0.089	0.119	0.126	0.048	0.052	0.077	0.105	0.111	0.130	0.142	0.216	0.304	0.323
26	0.064	0.069	0.096	0.128	0.137	0.052	0.057	0.084	0.115	0.121	0.149	0.163	0.248	0.349	0.370
27	0.067	0.073	0.103	0.139	0.148	0.057	0.062	0.092	0.127	0.134	0.167	0.183	0.278	0.392	0.416
28	0.071	0.077	0.110	0.150	0.160	0.062	0.067	0.101	0.140	0.147	0.183	0.201	0.308	0.435	0.462
29	0.074	0.081	0.117	0.161	0.173	0.065	0.072	0.109	0.152	0.161	0.196	0.216	0.336	0.479	0.510
30	0.076	0.084	0.124	0.174	0.187	0.068	0.075	0.116	0.162	0.172	0.208	0.230	0.363	0.524	0.558
31	0.078	0.086	0.131	0.188	0.203	0.070	0.078	0.122	0.172	0.183	0.217	0.241	0.390	0.572	0.611
32	0.079	0.088	0.139	0.203	0.220	0.072	0.080	0.128	0.183	0.194	0.223	0.251	0.417	0.623	0.667

GW, gestational weeks; p, percentile.

### Corpus callosum area

Reference curves for each of the five CC subdivisions were constructed using various distributions (Figures 5 and 6). The BCPE distribution was utilized for fetal CC total area (Table S3). The BCCG distribution was used for the fetal CC anterior one‐third, isthmus and splenium areas. Normal distribution and BCT distribution were used for the fetal CC anterior midbody area and posterior midbody area, respectively. The fetal CC area showed distinct growth patterns across different subdivisions. The percentile curve for fetal CC total area demonstrated a steady increase from 18 to 32 weeks (Figure 5). The anterior one‐third area increased sharply from 18 to 25 weeks, and then maintained stable growth until 32 weeks (Figure 6b, Table 1). Similarly to the growth trend in body thickness, anterior midbody, posterior midbody and isthmus areas showed a rapid increase from 18 to 23 weeks, followed by only a slight increase in area thereafter (Figure 6d–f, Table 1). The splenium area exhibited a steady increase from 18 to 32 weeks (Figure 6c, Table 1). Notably, the splenium demonstrated the most pronounced absolute area growth among all subdivisions, reaching a median of approximately 0.40 cm^2^ by 32 weeks, which is approximately three‐fold the area of the midbody regions (approximately 0.14 cm^2^ at the same GA). This posterior‐predominant growth pattern is consistent with the posterior‐to‐anterior maturation gradient of callosal fibers reported previously in postnatal imaging studies^14^, ^15^.

**Figure 5:**
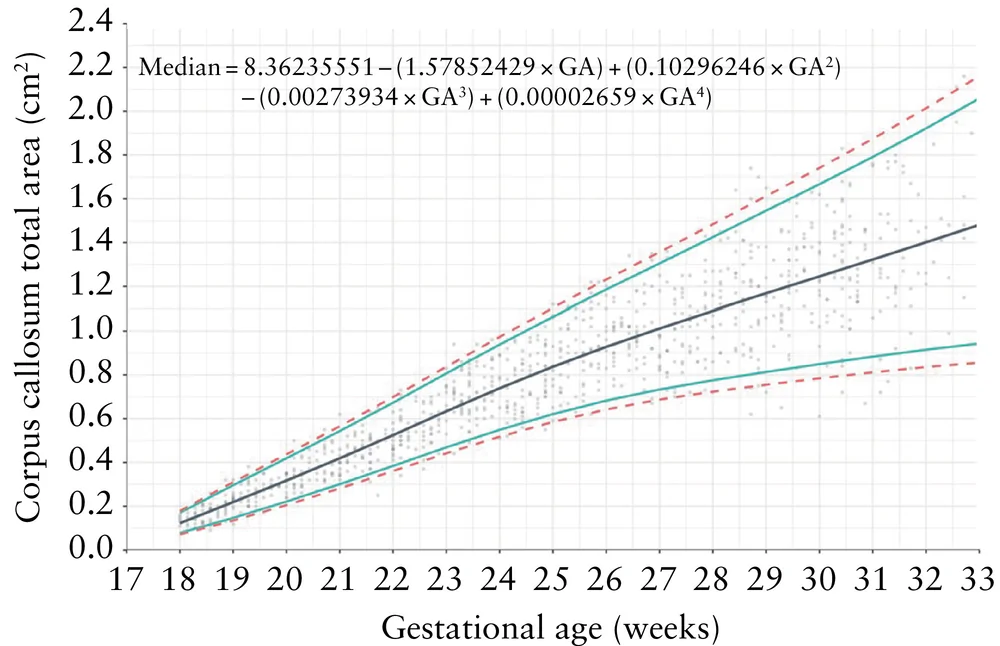
Percentile curves of fetal corpus callosum total area on non‐volume contrast imaging. 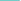, 5^th^ and 95^th^ percentiles; 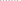, 2.5^th^ and 97.5^th^ percentiles; 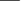, median; GA, gestational age.

**Figure 6:**
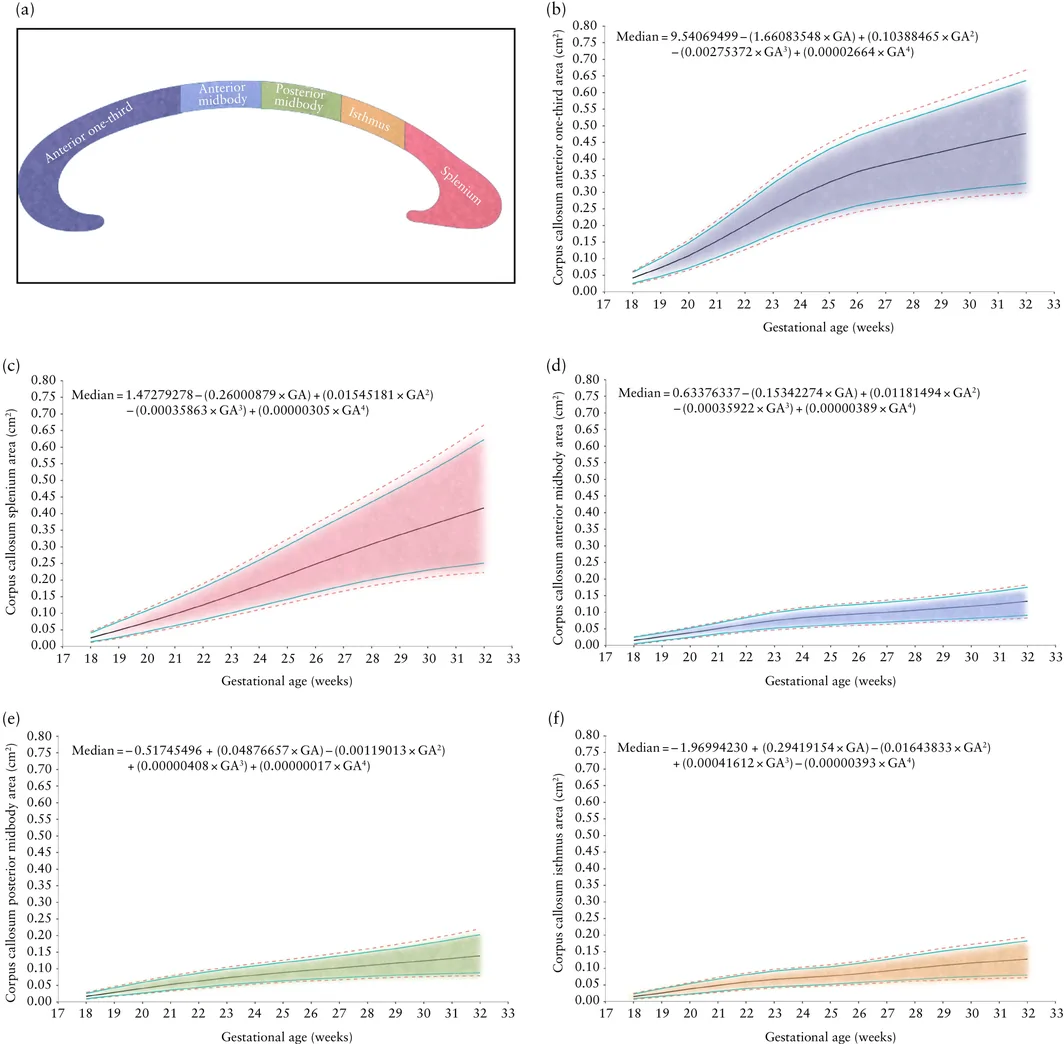
Regional growth patterns of fetal corpus callosum subdivisions at 18–32 weeks' gestation. (a) Schematic of the Witelson five‐subdivision algorithm, with color coding for the anterior one‐third (dark blue), anterior midbody (light blue), posterior midbody (green), isthmus (orange) and splenium (pink) regions. (b–f) Percentile curves of the corpus callosum subdivision areas measured on non‐volume contrast imaging: (b) anterior one‐third area; (c) splenium area; (d) anterior midbody area; (e) posterior midbody area; and (f) isthmus area. 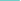, 5^th^ and 95^th^ percentiles; 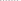, 2.5^th^ and 97.5^th^ percentiles; 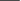, median; GA, gestational age.

### Intra‐ and interobserver reproducibility

The intraobserver reproducibility was good‐to‐excellent for fetal CC length, total area, genu, body and splenium thicknesses and all subdivision areas, with ICCs ranging from 0.838 (95% CI, 0.696–0.917) to 0.995 (95% CI, 0.990–0.998). The ICC for interobserver agreement varied depending on the fetal CC parameter, whereby the ICC showed good‐to‐excellent agreement for fetal CC length, total area, splenium thickness and all CC subdivision areas, while agreement was moderate for genu and body thicknesses (Table S4).

## DISCUSSION

### Principal findings

These percentile curves are intended to describe developmental distributions across gestation and should not be interpreted as validated clinical standards for defining abnormal development. Firstly, GA‐specific trajectories of fetal CC biometry were established, demonstrating distinct, non‐linear growth patterns in CC length, total area, genu, body and splenium thicknesses and the areas of each CC subdivision, with posterior‐predominant area expansion most pronounced in the splenium from 18 to 32 weeks. Overall, fetal CC biometry in terms of CC length, thickness and area demonstrated rapid growth between 18 and 23–25 weeks, followed by slower development extending to 32 weeks. Secondly, fetal CC biometric measurements obtained using non‐VCI and VCI modes were comparable.

### Results in the context of what is known

VCI projects a 1–10 mm volume slice onto a two‐dimensional (2D) screen, smoothing the speckle pattern by filling gaps with tissue information from surrounding layers, thereby offering clearer delineation of boundaries that are subtle on conventional ultrasound. This is supported by previous studies showing superior resolution of ultrasound images using VCI compared with 2D ultrasound^20^, ^21^, ^22^. In this study, we have shown comparable results between VCI and non‐VCI modalities for fetal CC measurements across gestation.

It should be emphasized that CC length in this study was obtained primarily as an internal methodological benchmark, confirming that our 3D transvaginal neurosonographic technique yielded data fully consistent with the recommended reference of Paladini *et al*.^5^, which is established as the gold standard for fetal CC length on transvaginal ultrasound. Against this validated background, the principal contribution of the present work lies in the description of fetal CC thickness and segmental area development across gestation, aspects that have not been systematically characterized during the fetal period. Restricting CC length to a methodological validation role in this study ensured that a single authoritative reference, namely that of Paladini *et al*.^5^, continues to serve the clinical community.

Regarding fetal CC thickness, our study reveals CC region‐specific, non‐linear growth patterns from 18 to 32 weeks, extending the current understanding of CC development^23^, ^24^. This aligns with studies reporting a two‐to‐three‐fold increase in the thickness of the CC during pregnancy^23^, ^24^. CC growth was shown previously to accelerate until 21–22 weeks, at which point it remains stable for the remainder of the pregnancy^23^. Our study refines this timeline by showing distinct growth patterns for different regions: the genu and body exhibited a rapid increase in thickness from 18 to 23 weeks, followed by more gradual growth to 32 weeks, while the splenium showed a prolonged period of sharp growth until 25 weeks, before growth appeared to slow. These patterns are consistent with earlier observations that CC structural formation is largely complete by around 21 weeks' gestation^25^. A postmortem immunohistochemical study of 46 fetal brains demonstrated that the CC area showed active growth between 20 and 26 postconceptional weeks, followed by a plateau between 27 and 33 postconceptional weeks^26^. These findings collectively support the assumption that cortical development undergoes a rapid increase in the second trimester, consistent with the period during which the fetal CC develops gradually along its longitudinal axis. The processes of axonal pruning and the reduction in interhemispheric connectivity during later stages of gestation are not addressed. These mechanisms may partly explain the observed plateau in CC growth trajectories and would provide valuable biological context for the imaging findings.

### Strengths and limitations

The main strength of this study is the establishment of fetal CC developmental trajectories of comprehensive CC biometry derived from 3D transvaginal ultrasound, supported by robust modeling and reproducibility analysis. Even in the absence of overt neurological abnormalities at birth, subtle neurodevelopmental or neuropsychiatric conditions may manifest later, and thus normal development cannot be fully guaranteed in all included cases. The low drop‐out rate reported in this study may be due to the predefined image‐quality criteria in the retrospective cohort. Consequently, the generalizability of the interpretation of fetal CC trajectories derived from this study may be limited. The reported trajectories are derived from a cross‐sectional cohort and therefore describe population‐level developmental patterns rather than individual growth trajectories. The study population was derived from a limited number of ethnic groups, and population‐specific variation in CC development cannot be excluded. Additionally, further work is required to evaluate the feasibility of extending the fetal CC trajectories beyond 32 weeks' gestation.

### Clinical and research implications

Our study has established trajectories of fetal CC development across gestation based on a five‐subdivision anatomical algorithm. The application of 3D ultrasound allowed for reconstruction of the fetal brain image using a multiplanar technique to easily visualize the CC, thereby significantly reducing scan difficulty when compared with the 2D approach. Overall reproducibility for most CC biometric measurements was good‐to‐excellent. However, genu thickness and body thickness measurements of the CC with moderate interobserver agreement should be interpreted with caution. Two methodological caveats apply. The method used to measure CC thickness was adapted from a postnatal MRI‐based methodology, with assessment of isthmus thickness not performed, and genu and body thicknesses demonstrated only moderate reliability. Witelson subdivisions use proportional, not anatomical, boundaries that vary with gestation. Thus values are descriptive, not clinically actionable, and are not ready for diagnostic use or counseling without longitudinal validation. These CC trajectories are not intended for use as a reference chart for isolated clinical decision‐making, particularly not for counseling regarding termination of pregnancy, until validated by long‐term neurodevelopmental outcome data. Moreover, isolated deviations from the normal reference range (e.g. reduced thickness or area in specific CC regions) may represent normal individual biological variation rather than pathology. In particular, the increasing standard deviation of CC thickness at later gestation probably reflects genuine biological variability rather than methodological imprecision. Whether normalization of CC thickness to head circumference or fronto‐occipital diameter would improve the GA correlation remains to be determined prospectively.

### Conclusions

We have characterized region‐specific non‐linear growth patterns in fetal CC development from 18 to 32 weeks' gestation, with posterior‐predominant area expansion most pronounced in the splenium. The clinical significance of regional CC measurements remains uncertain, and the present trajectories should be regarded as descriptions of normal developmental patterns rather than as reference charts for isolated clinical decision‐making. Longitudinal studies incorporating postnatal neurodevelopmental assessment are required to determine the prognostic value of these charts.

## Supporting information


**Table S1** Number of recruited cases for fetal corpus callosum biometry by gestational week (18–32 weeks).
**Table S2** Comparison of fetal corpus callosum biometry obtained using non‐volume contrast imaging and volume contrast imaging at 18–32 weeks' gestation.
**Table S3** Median, coefficient of variation, skewness and kurtosis for reference equations for corpus callosum biometric measurements using non‐volume contrast imaging.
**Table S4** Inter‐ and intraobserver reproducibility for obtaining fetal corpus callosum biometric parameters.

## Data Availability

The data that support the findings of this study are available from the corresponding author upon reasonable request.
