# Supplementary material for: Regional growth patterns of fetal corpus callosum at 18–32 weeks' gestation assessed by three‐dimensional transvaginal neurosonography
Source: Ultrasound Obstet Gynecol. 2026 Oct 21;68(5):636–46. doi: 10.1002/uog.70334 (PMC13730427; doi:10.1002/uog.70334)
Supplement: Supplementary file 1 [file UOG-68-636-s001.docx]

**Table S1 Number of recruited cases for fetal corpus callosum biometry by gestational week (18–32 weeks).**

| **Gestational age (weeks)** | **Numbers** |
| --- | --- |
| 18 | 58 |
| 19 | 60 |
| 20 | 63 |
| 21 | 63 |
| 22 | 70 |
| 23 | 83 |
| 24 | 79 |
| 25 | 77 |
| 26 | 70 |
| 27 | 72 |
| 28 | 67 |
| 29 | 62 |
| 30 | 72 |
| 31 | 40 |
| 32 | 20 |
| Total | 956 |

**Table S2 Comparison of fetal corpus callosum biometry obtained using non-volume contrast imaging and volume contrast imaging at 18–32 weeks’ gestation.**

| **Parameters at 18 weeks** | **Non-VCI CC Median (IQR)** | **VCI CC Median (IQR)** | **P value** | **Parameters at 19 weeks** | **Non-VCI CC Median (IQR)** | **VCI CC**  **Median (IQR)** | **P value** |
| --- | --- | --- | --- | --- | --- | --- | --- |
| **Length (cm)** | 1.28 (1.17, 1.42) | 1.33 (1.18, 1.45) | 0.661 | **Length (cm)** | 1.59 (1.50, 1.71) | 1.61 (1.50, 1.74) | 0.600 |
| **Total area (cm^2^)** | 0.16 (0.13, 0.19) | 0.15 (0.12, 0.19) | 0.498 | **Total area (cm^2^)** | 0.26 (0.22, 0.30) | 0.24 (0.20, 0.28) | 0.139 |
| **Genu thickness (cm)** | 0.13 (0.12, 0.15) | 0.13 (0.12, 0.15) | 0.734 | **Genu thickness (cm)** | 0.15 (0.14, 0.17) | 0.15 (0.13, 0.17) | 0.378 |
| **Body thickness (cm)** | 0.10 (0.08, 0.11) | 0.09 (0.08, 0.11) | 0.188 | **Body thickness (cm)** | 0.12 (0.11, 0.14) | 0.11 (0.10, 0.13) | 0.097 |
| **Splenium thickness (cm)** | 0.13 (0.12, 0.15) | 0.13 (0.11, 0.15) | 0.973 | **Splenium thickness (cm)** | 0.15 (0.13, 0.17) | 0.15 (0.14, 0.17) | 0.483 |
| **Anterior one-third area (cm^2^)** | 0.06 (0.05, 0.07) | 0.05 (0.04, 0.06) | 0.269 | **Anterior one-third area (cm^2^)** | 0.08 (0.08, 0.10) | 0.08 (0.07, 0.09) | 0.080 |
| **Anterior midbody area (cm^2^)** | 0.02 (0.02, 0.02) | 0.02 (0.02, 0.02) | 0.842 | **Anterior midbody area (cm^2^)** | 0.03 (0.03, 0.04) | 0.03 (0.03, 0.03) | 0.645 |
| **Posterior midbody area (cm^2^)** | 0.02 (0.02, 0.02) | 0.02 (0.02, 0.03) | 0.830 | **Posterior midbody area (cm^2^)** | 0.03 (0.03, 0.04) | 0.03 (0.03, 0.04) | 0.292 |
| **Isthmus area (cm^2^)** | 0.02 (0.02, 0.02) | 0.02 (0.01, 0.02) | 0.284 | **Isthmus area (cm^2^)** | 0.03 (0.03, 0.04) | 0.03 (0.02, 0.04) | 0.359 |
| **Splenium area (cm^2^)** | 0.04 (0.03, 0.04) | 0.04 (0.03, 0.04) | 0.869 | **Splenium area (cm^2^)** | 0.06 (0.05, 0.07) | 0.06 (0.04, 0.07) | 0.555 |
| **Parameters at 20 weeks** | **Non-VCI CC Median (IQR)** | **VCI CC Median (IQR)** | **P value** | **Parameters at 21 weeks** | **Non-VCI CC Median (IQR)** | **VCI CC Median (IQR)** | **P value** |
| **Length (cm)** | 1.91 (1.75, 2.05) | 1.91 (1.79, 2.04) | 0.762 | **Length (cm)** | 2.20 (2.09, 2.30) | 2.22 (2.08, 2.32) | 0.738 |
| **Total area (cm^2^)** | 0.35 (0.31, 0.42) | 0.34 (0.29, 0.40) | 0.413 | **Total area (cm^2^)** | 0.44 (0.40, 0.50) | 0.46 (0.40, 0.50) | 0.940 |
| **Genu thickness (cm)** | 0.18 (0.16, 0.20) | 0.17 (0.16, 0.19) | 0.231 | **Genu thickness (cm)** | 0.19 (0.18, 0.22) | 0.21 (0.18, 0.23) | 0.189 |
| **Body thickness (cm)** | 0.13 (0.12, 0.15) | 0.13 (0.12, 0.15) | 0.557 | **Body thickness (cm)** | 0.15 (0.14, 0.17) | 0.15 (0.13, 0.17) | 0.896 |
| **Splenium thickness (cm)** | 0.18 (0.16, 0.20) | 0.18 (0.16, 0.20) | 0.75 | **Splenium thickness (cm)** | 0.20 (0.18, 0.24) | 0.21 (0.19, 0.24) | 0.670 |
| **Anterior one-third area (cm^2^)** | 0.12 (0.11, 0.15) | 0.12 (0.10, 0.13) | 0.173 | **Anterior one-third area (cm^2^)** | 0.17 (0.15, 0.19) | 0.17 (0.15, 0.20) | 0.439 |
| **Anterior midbody area (cm^2^)** | 0.04 (0.04, 0.05) | 0.04 (0.04, 0.05) | 0.564 | **Anterior midbody area (cm^2^)** | 0.06 (0.05, 0.06) | 0.06 (0.05, 0.06) | 0.945 |
| **Posterior midbody area (cm^2^)** | 0.05 (0.04, 0.05) | 0.04 (0.04, 0.05) | 0.497 | **Posterior midbody area (cm^2^)** | 0.06 (0.05, 0.07) | 0.05 (0.05, 0.06) | 0.085 |
| **Isthmus area (cm^2^)** | 0.04 (0.04, 0.05) | 0.04 (0.04, 0.05) | 0.541 | **Isthmus area (cm^2^)** | 0.05 (0.05, 0.06) | 0.05 (0.04, 0.06) | 0.523 |
| **Splenium area (cm^2^)** | 0.08 (0.07, 0.11) | 0.08 (0.07, 0.10) | 0.935 | **Splenium area (cm^2^)** | 0.11 (0.09, 0.12) | 0.11 (0.09, 0.12) | 0.992 |
| **Parameters at 22 weeks** | **Non-VCI CC Median (IQR)** | **VCI CC Median (IQR)** | **P value** | **Parameters at 23 weeks** | **Non-VCI CC Median (IQR)** | **VCI CC Median (IQR)** | **P value** |
| **Length (cm)** | 2.52 (2.34, 2.61) | 2.50 (2.35, 2.60) | 0.723 | **Length (cm)** | 2.77 (2.63, 2.94) | 2.81 (2.66, 2.94) | 0.546 |
| **Total area (cm^2^)** | 0.53 (0.48, 0.61) | 0.53 (0.44, 0.62) | 0.601 | **Total area (cm^2^)** | 0.72 (0.64, 0.80) | 0.72 (0.64, 0.80) | 0.859 |
| **Genu thickness (cm)** | 0.24 (0.21, 0.25) | 0.22 (0.20, 0.25) | 0.487 | **Genu thickness (cm)** | 0.28 (0.25, 0.30) | 0.27 (0.25, 0.30) | 0.930 |
| **Body thickness (cm)** | 0.16 (0.14, 0.18) | 0.16 (0.14, 0.18) | 0.609 | **Body thickness (cm)** | 0.18 (0.16, 0.20) | 0.17 (0.16, 0.20) | 0.268 |
| **Splenium thickness (cm)** | 0.24 (0.21, 0.26) | 0.23 (0.21, 0.25) | 0.475 | **Splenium thickness (cm)** | 0.28 (0.26, 0.30) | 0.28 (0.25, 0.31) | 0.441 |
| **Anterior one-third area (cm^2^)** | 0.21 (0.19, 0.23) | 0.21 (0.18, 0.23) | 0.735 | **Anterior one-third area (cm^2^)** | 0.27 (0.25, 0.32) | 0.28 (0.24, 0.31) | 0.635 |
| **Anterior midbody area (cm^2^)** | 0.07 (0.06, 0.08) | 0.06 (0.06, 0.07) | 0.285 | **Anterior midbody area (cm^2^)** | 0.08 (0.07, 0.10) | 0.08 (0.07, 0.09) | 0.239 |
| **Posterior midbody area (cm^2^)** | 0.07 (0.06, 0.08) | 0.07 (0.06, 0.08) | 0.499 | **Posterior midbody area (cm^2^)** | 0.08 (0.08, 0.09) | 0.08 (0.07, 0.09) | 0.211 |
| **Isthmus area (cm^2^)** | 0.06 (0.05, 0.07) | 0.06 (0.05, 0.07) | 0.164 | **Isthmus area (cm^2^)** | 0.07 (0.06, 0.09) | 0.07 (0.06, 0.08) | 0.962 |
| **Splenium area (cm^2^)** | 0.13 (0.11, 0.14) | 0.13 (0.11, 0.15) | 0.971 | **Splenium area (cm^2^)** | 0.17 (0.15, 0.20) | 0.17 (0.15, 0.20) | 0.710 |
| **Parameters at 24 weeks** | **Non-VCI CC Median (IQR)** | **VCI CC Median (IQR)** | **P value** | **Parameters at 25 weeks** | **Non-VCI CC Median (IQR)** | **VCI CC Median (IQR)** | **P value** |
| **Length (cm)** | 3.01 (2.82, 3.23) | 3.01 (2.82, 3.22) | 0.816 | **Length (cm)** | 3.20 (3.05, 3.36) | 3.22 (3.01, 3.36) | 0.877 |
| **Total area (cm^2^)** | 0.78 (0.66, 0.88) | 0.76 (0.66, 0.86) | 0.825 | **Total area (cm^2^)** | 0.90 (0.78, 1.02) | 0.88 (0.77, 0.99) | 0.514 |
| **Genu thickness (cm)** | 0.26 (0.23, 0.30) | 0.24 (0.22, 0.28) | 0.226 | **Genu thickness (cm)** | 0.28 (0.25, 0.31) | 0.27 (0.25, 0.32) | 0.679 |
| **Body thickness (cm)** | 0.17 (0.16, 0.19) | 0.17 (0.15, 0.19) | 0.223 | **Body thickness (cm)** | 0.18 (0.16, 0.20) | 0.17 (0.16, 0.19) | 0.135 |
| **Splenium thickness (cm)** | 0.35 (0.31, 0.38) | 0.32 (0.30, 0.36) | 0.138 | **Splenium thickness (cm)** | 0.37 (0.31, 0.42) | 0.35 (0.30, 0.40) | 0.184 |
| **Anterior one-third area (cm^2^)** | 0.30 (0.26, 0.33) | 0.29 (0.26, 0.33) | 0.849 | **Anterior one-third area (cm^2^)** | 0.35 (0.31, 0.40) | 0.35 (0.30, 0.39) | 0.524 |
| **Anterior midbody area (cm^2^)** | 0.08 (0.08, 0.09) | 0.08 (0.07, 0.09) | 0.118 | **Anterior midbody area (cm^2^)** | 0.09 (0.08, 0.10) | 0.09 (0.08, 0.10) | 0.123 |
| **Posterior midbody area (cm^2^)** | 0.08 (0.07, 0.09) | 0.08 (0.07, 0.09) | 0.213 | **Posterior midbody area (cm^2^)** | 0.09 (0.08, 0.10) | 0.09 (0.08, 0.10) | 0.071 |
| **Isthmus area (cm^2^)** | 0.07 (0.06, 0.08) | 0.07 (0.06, 0.08) | 0.184 | **Isthmus area (cm^2^)** | 0.08 (0.07, 0.09) | 0.08 (0.07, 0.09) | 0.506 |
| **Splenium area (cm^2^)** | 0.19 (0.16, 0.23) | 0.19 (0.16, 0.22) | 0.692 | **Splenium area (cm^2^)** | 0.23 (0.20, 0.27) | 0.22 (0.19, 0.27) | 0.672 |
| **Parameters at 26 weeks** | **Non-VCI CC Median (IQR)** | **VCI CC Median (IQR)** | **P value** | **Parameters at 27 weeks** | **Non-VCI CC Median (IQR)** | **VCI CC Median (IQR)** | **P value** |
| **Length (cm)** | 3.20 (3.05, 3.36) | 3.22 (3.01, 3.36) | 0.877 | **Length (cm)** | 3.54 (3.32, 3.69) | 3.52 (3.36, 3.75) | 0.975 |
| **Total area (cm^2^)** | 0.90 (0.78, 1.02) | 0.88 (0.77, 0.99) | 0.514 | **Total area (cm^2^)** | 1.00 (0.86, 1.14) | 0.98 (0.85, 1.08) | 0.353 |
| **Genu thickness (cm)** | 0.28 (0.25, 0.31) | 0.27 (0.25, 0.32) | 0.679 | **Genu thickness (cm)** | 0.27 (0.26, 0.32) | 0.28 (0.25, 0.31) | 0.804 |
| **Body thickness (cm)** | 0.18 (0.16, 0.20) | 0.17 (0.16, 0.19) | 0.135 | **Body thickness (cm)** | 0.17 (0.16, 0.18) | 0.17 (0.15, 0.19) | 0.181 |
| **Splenium thickness (cm)** | 0.37 (0.31, 0.42) | 0.35 (0.30, 0.40) | 0.184 | **Splenium thickness (cm)** | 0.38 (0.35, 0.43) | 0.36 (0.33, 0.41) | 0.088 |
| **Anterior one-third area (cm^2^)** | 0.35 (0.31, 0.40) | 0.35 (0.30, 0.39) | 0.524 | **Anterior one-third area (cm^2^)** | 0.38 (0.34, 0.42) | 0.38 (0.33, 0.43) | 0.831 |
| **Anterior midbody area (cm^2^)** | 0.09 (0.08, 0.10) | 0.09 (0.08, 0.10) | 0.123 | **Anterior midbody area (cm^2^)** | 0.10 (0.09, 0.11) | 0.09 (0.08, 0.10) | 0.164 |
| **Posterior midbody area (cm^2^)** | 0.09 (0.08, 0.10) | 0.09 (0.08, 0.10) | 0.071 | **Posterior midbody area (cm^2^)** | 0.10 (0.09, 0.11) | 0.09 (0.09, 0.10) | 0.126 |
| **Isthmus area (cm^2^)** | 0.08 (0.07, 0.09) | 0.08 (0.07, 0.09) | 0.506 | **Isthmus area (cm^2^)** | 0.09 (0.08, 0.10) | 0.09 (0.07, 0.10) | 0.433 |
| **Splenium area (cm^2^)** | 0.23 (0.20, 0.27) | 0.22 (0.19, 0.27) | 0.672 | **Splenium area (cm^2^)** | 0.29 (0.24, 0.32) | 0.28 (0.23, 0.32) | 0.821 |
| **Parameters at 28 weeks** | **Non-VCI CC Median (IQR)** | **VCI CC Median (IQR)** | **P value** | **Parameters at 29 weeks** | **Non-VCI CC Median (IQR)** | **VCI CC Median (IQR)** | **P value** |
| **Length (cm)** | 3.72 (3.52, 3.86) | 3.68 (3.53, 3.85) | 0.933 | **Length (cm)** | 3.80(3.61, 3.99) | 3.77(3.59, 4.00) | 0.968 |
| **Total area (cm^2^)** | 1.15 (0.93, 1.28) | 1.08 (0.94, 1.23) | 0.313 | **Total area (cm^2^)** | 1.25(1.08, 1.38) | 1.19(1.05, 1.34) | 0.162 |
| **Genu thickness (cm)** | 0.30 (0.28, 0.33) | 0.30 (0.27, 0.32) | 0.423 | **Genu thickness (cm)** | 0.30(0.28, 0.33) | 0.30(0.27, 0.33) | 0.918 |
| **Body thickness (cm)** | 0.19 (0.17, 0.21) | 0.19 (0.16, 0.20) | 0.078 | **Body thickness (cm)** | 0.19(0.17, 0.21) | 0.18(0.16, 0.20) | 0.173 |
| **Splenium thickness (cm)** | 0.43 (0.36, 0.46) | 0.41 (0.36, 0.44) | 0.357 | **Splenium thickness (cm)** | 0.42(0.39, 0.46) | 0.41(0.39, 0.44) | 0.092 |
| **Anterior one-third area (cm^2^)** | 0.41 (0.36, 0.46) | 0.41 (0.36, 0.46) | 0.993 | **Anterior one-third area (cm^2^)** | 0.45(0.38, 0.48) | 0.41(0.37, 0.46) | 0.21 |
| **Anterior midbody area (cm^2^)** | 0.11 (0.10, 0.13) | 0.11 (0.09, 0.11) | 0.100 | **Anterior midbody area (cm^2^)** | 0.11(0.10, 0.13) | 0.10(0.10, 0.12) | 0.105 |
| **Posterior midbody area (cm^2^)** | 0.11 (0.10, 0.13) | 0.11 (0.10, 0.12) | 0.452 | **Posterior midbody area (cm^2^)** | 0.12(0.11, 0.14) | 0.12(0.10, 0.13) | 0.177 |
| **Isthmus area (cm^2^)** | 0.11 (0.10, 0.13) | 0.11 (0.09, 0.12) | 0.585 | **Isthmus area (cm^2^)** | 0.11(0.10, 0.13) | 0.11(0.10, 0.13) | 0.478 |
| **Splenium area (cm^2^)** | 0.32 (0.29, 0.40) | 0.33 (0.28, 0.40) | 0.817 | **Splenium area (cm^2^)** | 0.38(0.32, 0.42) | 0.37(0.31, 0.41) | 0.3 |
| **Parameters at 30 weeks** | **Non-VCI CC Median (IQR)** | **VCI CC Median (IQR)** | **P value** | **Parameters at 31 weeks** | **Non-VCI CC Median (IQR)** | **VCI CC Median (IQR)** | **P value** |
| **Length (cm)** | 3.95 (3.72, 4.08) | 3.92 (3.77, 4.12) | 0.992 | **Length (cm)** | 3.94 (3.83, 4.20) | 3.89 (3.79, 4.17) | 0.672 |
| **Total area** **(cm^2^)** | 1.24 (1.12, 1.44) | 1.21 (1.07, 1.36) | 0.218 | **Total area (cm^2^)** | 1.27 (1.14, 1.55) | 1.23 (0.99, 1.45) | 0.204 |
| **Genu thickness (cm)** | 0.31 (0.28, 0.34) | 0.30 (0.28, 0.34) | 0.511 | **Genu thickness (cm)** | 0.32 (0.28, 0.37) | 0.31 (0.27, 0.35) | 0.365 |
| **Body thickness (cm)** | 0.19 (0.17, 0.21) | 0.18 (0.17, 0.21) | 0.276 | **Body thickness (cm)** | 0.20 (0.17, 0.23) | 0.20 (0.16, 0.22) | 0.229 |
| **Splenium thickness (cm)** | 0.44 (0.36, 0.48) | 0.42 (0.36, 0.46) | 0.386 | **Splenium thickness (cm)** | 0.46 (0.39, 0.50) | 0.42 (0.37, 0.47) | 0.068 |
| **Anterior one-third area (cm^2^)** | 0.45 (0.40, 0.50) | 0.43 (0.38, 0.47) | 0.112 | **Anterior one-third area (cm^2^)** | 0.49 (0.41, 0.58) | 0.45 (0.39, 0.53) | 0.321 |
| **Anterior midbody area (cm^2^)** | 0.12 (0.10, 0.13) | 0.11 (0.10, 0.12) | 0.120 | **Anterior midbody area (cm^2^)** | 0.14 (0.11, 0.15) | 0.12 (0.10, 0.14) | 0.140 |
| **Posterior midbody area (cm^2^)** | 0.12 (0.11, 0.14) | 0.12 (0.10, 0.13) | 0.264 | **Posterior midbody area (cm^2^)** | 0.14 (0.11, 0.16) | 0.13 (0.10, 0.14) | 0.395 |
| **Isthmus area (cm^2^)** | 0.11 (0.10, 0.14) | 0.11 (0.10, 0.13) | 0.704 | **Isthmus area (cm^2^)** | 0.13 (0.10, 0.16) | 0.11 (0.09, 0.15) | 0.325 |
| **Splenium area (cm^2^)** | 0.37 (0.30, 0.41) | 0.35 (0.31, 0.40) | 0.136 | **Splenium area (cm^2^)** | 0.41 (0.32, 0.47) | 0.36 (0.30, 0.44) | 0.231 |
| **Parameters at 32 weeks** | **Non-VCI CC Median (IQR)** | **VCI CC Median (IQR)** | **P value** |  |  |  |  |
| **Length (cm)** | 4.22 (4.00, 4.42) | 4.22 (4.02, 4.38) | 0.871 |  |  |  |  |
| **Total area (cm^2^)** | 1.44 (1.30, 1.67) | 1.36 (1.22, 1.67) | 0.570 |  |  |  |  |
| **Genu thickness (cm)** | 0.34 (0.30, 0.38) | 0.34 (0.31, 0.37) | 0.935 |  |  |  |  |
| **Body thickness (cm)** | 0.22 (0.20, 0.26) | 0.21 (0.18, 0.23) | 0.174 |  |  |  |  |
| **Splenium thickness (cm)** | 0.49 (0.42, 0.53) | 0.48 (0.43, 0.50) | 0.745 |  |  |  |  |
| **Anterior one-third area (cm^2^)** | 0.52 (0.46, 0.56) | 0.48 (0.43, 0.58) | 0.516 |  |  |  |  |
| **Anterior midbody area (cm^2^)** | 0.15 (0.13, 0.18) | 0.14 (0.11, 0.17) | 0.276 |  |  |  |  |
| **Posterior midbody area (cm^2^)** | 0.16 (0.14, 0.19) | 0.15 (0.12, 0.18) | 0.335 |  |  |  |  |
| **Isthmus area (cm^2^)** | 0.15 (0.13, 0.17) | 0.15 (0.12, 0.16) | 0.745 |  |  |  |  |
| **Splenium area (cm^2^)** | 0.46 (0.39, 0.56) | 0.46 (0.38, 0.56) | 0.745 |  |  |  |  |

**Table S3 Median, coefficient of variation and skewness for reference equations for corpus callosum biometric measurements using non-volume contrast imaging.**

| **Parameter** | **Corpus callosum length (cm)** | **Corpus callosum total area (cm^2^)** |
| --- | --- | --- |
| Distribution | Normal distribution | BCPE distribution |
| Median | 14.81412732-3.20422693 × GA + 0.23400251 × GA^2 -0.00666237 × GA^3 + 0.00006724 × GA^4 | 8.36235551-1.57852429 × GA + 0.10296246 × GA^2 -0.00273934 × GA^3 + 0.00002659 × GA^4 |
| CV | 0.65603560-0.09962413 × GA + 0.00632998 × GA^2 -0.00015980 × GA^3 + 0.00000153 × GA^4 | 1.49904311-0.10914878 × GA + 0.00130118 × GA^2 + 0.00007328 × GA^3 -0.00000145 × GA^4 |
| Skewness | - | 0.81497764 |
| Kurtosis | - | 2.40024855 |
|  | **Corpus callosum genu thickness (cm)** | **Corpus callosum body thickness (cm)** |
| Distribution | Normal distribution | BCCG distribution |
| Median | 4.30137116-0.79879175 × GA + 0.05382174 × GA^2 -0.00152157 × GA^3 + 0.00001557 × GA^4 | -1.50585624+ 0.15080887 * GA -0.00298201 * GA^2 -0.00005875 * GA^3 + 0.00000178 * GA^4 |
| CV | 3.13950383-0.53180992 × GA + 0.03326246 × GA^2 -0.00090288 × GA^3 + 0.00000901 × GA^4 | -0.22533421+ 0.10864485 * GA -0.00869987 * GA^2 + 0.00027057 * GA^3 -0.00000291 * GA^4 |
| Skewness | - | 0.62473274 |
| Kurtosis | - | - |
|  | **Corpus callosum splenium thickness (cm)** | **Corpus callosum anterior one-third area(cm^2^)** |
| Distribution | BCPE distribution | BCCG distribution |
| Median | 15.61908411-2.59735621 × GA + 0.15841106 × GA^2 -0.00416308 × GA^3 + 0.00004019 × GA^4 | 9.54069499-1.66083548 × GA + 0.10388465 × GA^2 -0.00275372 × GA^3 + 0.00002664 × GA^4 |
| CV | 0.15922557+ 0.00019314 × GA -0.00000131 × GA^2 + 0.00000010 × GA^3 -0.00000000 × GA^4 | 0.87043009-0.04480072 × GA -0.00014848 × GA^2 + 0.00005275 × GA^3 -0.00000078 × GA^4 |
| Skewness | 1.16273431 | 0.81794287 |
| Kurtosis | 2.3992005 | - |
|  | **Corpus callosum anterior midbody area (cm^2^)** | **Corpus callosum posterior midbody area (cm^2^)** |
| Distribution | Normal distribution | BCT distribution |
| Median | 0.63376337-0.15342274 × GA + 0.01181494 × GA^2 -0.00035922 × GA^3 + 0.00000389 × GA^4 | -0.51745496+ 0.04876657 × GA -0.00119013 × GA^2 + 0.00000408 × GA^3 + 0.00000017 × GA^4 |
| CV | 0.37914872-0.06876050 × GA + 0.00454084 × GA^2 -0.00012792 × GA^3 + 0.00000132 × GA^4 | 1.84809151-0.17282096 × GA + 0.00612641 × GA^2 -0.00008920 × GA^3 + 0.00000054 × GA^4 |
| Skewness | - | 0.39029424 |
| Kurtosis | - | 10.93342476 |
|  | **Corpus callosum isthmus area (cm^2^)** | **Corpus callosum splenium area (cm^2^)** |
| Distribution | BCCG distribution | BCCG distribution |
| Median | -1.96994230+ 0.29419154 × GA -0.01643833 × GA^2 + 0.00041612 × GA^3 -0.00000393 × GA^4 | 1.47279278-0.26000879 × GA + 0.01545181 × GA^2 -0.00035863 × GA^3 + 0.00000305 × GA^4 |
| CV | 2.97624369-0.34505474 × GA + 0.01595880 × GA^2 -0.00032831 × GA^3 + 0.00000260 × GA^4 | 5.96767075-0.82686312 × GA + 0.04494396 × GA^2 -0.00109690 × GA^3 + 0.00001019 × GA^4 |
| Skewness | 0.64474725 | 0.53165957 |
| Kurtosis | - | - |

CV: coefficient of variation

**Table S4 Inter- and intraobserver reproducibility for obtaining all fetal corpus callosum biometric parameters.**

| **Parameter** | **Interobserver** | **Intraobserver** |
| --- | --- | --- |
|  | **ICC (95% CI)** | **ICC (95% CI)** |
| **Length** | 0.983 (0.966 to 0.992) | 0.995 (0.990 to 0.998) |
| **Total area** | 0.946 (0.892 to 0.973) | 0.988 (0.975 to 0.994) |
| **Genu thickness** | 0.749 (0.442 to 0.883) | 0.955 (0.910 to 0.978) |
| **Body thickness** | 0.703 (0.469 to 0.844) | 0.930 (0.863 to 0.965) |
| **Splenium thickness** | 0.860 (0.731 to 0.929) | 0.936 (0.873 to 0.968) |
| **Anterior one-third area** | 0.940 (0.806 to 0.976) | 0.967 (0.934 to 0.984) |
| **Anterior midbody area** | 0.893 (0.792 to 0.946) | 0.870 (0.752 to 0.934) |
| **Posterior midbody area** | 0.929 (0.860 to 0.965) | 0.927 (0.818 to 0.968) |
| **Isthmus area** | 0.793 (0.619 to 0.893) | 0.838 (0.696 to 0.917) |
| **Splenium area** | 0.882 (0.772 to 0.940) | 0.936 (0.873 to 0.968) |

Agreement was classified as excellent (ICC, ≥0.90), good (ICC, 0.75 to <0.90), moderate (ICC, 0.50 to <0.75), or poor (ICC, <0.50).
